# Endocrine and metabolic complications in a national cohort of Slovene children and adolescents with Duchenne muscular dystrophy: real-world criteria for transition to vamorolone therapy

**DOI:** 10.3389/fendo.2025.1697907

**Published:** 2025-12-05

**Authors:** Sončka Jazbinšek, Eva Vrščaj, Jasna Šuput Omladič, Tita Butenko, Tanja Loboda, Primož Kotnik, Damjan Osredkar

**Affiliations:** 1 Department of Endocrinology, Diabetes and Metabolism, University Children’s Hospital, University Medical Center Ljubljana, Ljubljana, Slovenia; 2 Department of Pediatric Neurology, University Children’s Hospital, University Medical Centre Ljubljana, Ljubljana, Slovenia; 3 Faculty of Medicine, University of Ljubljana, Ljubljana, Slovenia

**Keywords:** Duchenne muscular dystrophy, corticosteroid treatment, vamorolone, bone health, linear growth

## Abstract

**Aims:**

To assess the prevalence of endocrine/metabolic disorders among pediatric Duchenne muscular dystrophy (DMD) patients and identify individuals who would benefit from the new corticosteroid treatment available, vamorolone.

**Methods:**

A national pediatric cohort of DMD patients followed at University Children’s Hospital, University Medical Center Ljubljana in June 2025 was included in the study. The presence of endocrine/metabolic disorders was determined by clinical examination, laboratory data, and imaging at the last annual multidisciplinary evaluation.

**Results:**

A total of 21 patients (average age 11.1, range 4.6-16.7 yrs) were included in the study. Two patients were corticosteroid-naive, the rest treated with deflazacort (average treatment duration 5.4, range 1.1-11.3 yrs). At the time of final follow-up, 90% were diagnosed with an endocrine and/or metabolic disorder. Short stature was present in 67% of patients, a decline in height standard deviation score (SDS) since the initiation of corticosteroid therapy was observed, -2.37 SDS on average. Based on body composition data (fat % SDS), 57% were classified as obese. Lipid abnormalities were detected in 76% of patients. Insulin resistance (determined by HOMA-IR) was increased in 9.5% of patients; type 2 diabetes was not detected.

**Conclusion:**

Our findings demonstrate a high prevalence of endocrine and metabolic disturbances among patients with DMD. In light of emerging evidence on the potential benefits of vamorolone - particularly regarding bone health and growth - we identified a subgroup of patients most likely to benefit from its use. We propose that ambulatory, non-corticosteroid naive patients with pathological fractures, markedly reduced bone density, short stature, or significant growth deceleration could be considered for the therapeutic transition. Accordingly, we established national clinical criteria to guide individualized therapeutic transitions, aimed at optimizing clinical outcomes and ensuring efficient allocation of healthcare resources.

## Introduction

Duchenne muscular dystrophy (DMD) is a recessive X-linked neuromuscular disorder with an estimated incidence of 1 in 3500 – 5000, caused by mutations in the dystrophin gene (1, 2). These mutations result in absent or insufficient levels of functional dystrophin, leading to progressive muscle degeneration and weakness, disability, and a shortened life expectancy (3). As DMD is a multisystem disease, other organ systems besides muscles are also affected by disease progression. Respiratory impairment and cardiomyopathy are usually responsible for the premature death of untreated patients in their 30s; however, life expectancy has increased with evolving standards of care (3, 4).

The mainstream therapy for DMD is daily corticosteroid use, which is usually initiated between 4–6 years of age and improves muscle strength, slows muscle wasting, prolongs ambulation, improves cardiac function, decelerates the decline of respiratory function, decreases the need for scoliosis surgery, and prolongs survival (5, 6). However, corticosteroid treatment is associated with significant endocrine and metabolic side effects, which include stunted growth, decreased bone mineral density, delayed puberty, and glucose intolerance (3, 4). The psychosocial burden of these complications should not be underestimated (7). The significance of complications and their burden vary between individuals and over time. The side effects of corticosteroid use can progress over time, and specific interventions can mitigate some, while others may be more difficult to manage (8).

A new corticosteroid, vamorolone, was approved in October 2023 for the treatment of DMD in patients 2 years of age and older in the USA and received a positive opinion in the European Union in October 2023 for the treatment of DMD in patients 4 years of age and older (9) and now presents a third treatment option in addition to prednisolone and deflazacort in European countries. Classified as a dissociative corticosteroid, this drug exhibits anti-inflammatory properties comparable to prednisolone and deflazacort. However, its mechanism of action differs significantly; it antagonizes the mineralocorticoid receptor and lacks affinity for the glucocorticoid response element. This unique profile is thought to be responsible for a reduced side-effect burden compared to traditional corticosteroids (10, 11). To date, only data on the short-term use in ambulatory patients have been published. The results of its use have shown similar efficacy in retaining motor function as prednisolone while reducing some of the side effects linked to bone turnover, fracture rate, and linear growth (12–14). No clinical data are available regarding its potential cardioprotective benefits, effects on pubertal development, metabolic effects, effects on the respiratory system, or on cataract development. There are also no data on its use in non-ambulatory patients. The long-term efficacy and side effect profile of vamorolone remain to be elucidated.

With the recent availability of vamorolone, we sought to characterize the prevalence and severity of endocrine and metabolic complications within our cohort of patients with DMD. The data collected on these comorbidities together with anthropometric data may provide critical clinical insights, informing patient selection for a therapeutic transition to vamorolone and potentially mitigating adverse effects associated with conventional corticosteroid regimens. We propose criteria that could be helpful in selecting patients for switch from prednisolone/deflazacort to vamorolone treatment. This targeted approach could optimize patient outcomes and enhance overall quality of life.

## Methods

### Study population

In this observational cohort study, we collected data from a cohort of patients with a genetically confirmed diagnosis of DMD who were treated at the Department of Child, Adolescent, and Developmental Neurology of the University Children’s Hospital, Ljubljana, Slovenia. This is the only tertiary pediatric neurology center in Slovenia, and all pediatric and adolescent patients with DMD in the country are being followed up at the same department. DMD patients were evaluated at least once per year by a multidisciplinary team. Data were collected from electronic medical records between September 2022 and May 2025. When the values of the analyzed parameters at different time points were available, the latest data were used for analysis. This study was approved by the National Medical Ethics Committee of Slovenia (0120-160/2016-2).

### Multidisciplinary evaluation

As part of the two-day yearly evaluation at our department, various specialists from the multidisciplinary team for neuromuscular diseases examined the patients. The team consisted of neurologists, endocrinologists, pulmonologists, cardiologists, gastroenterologists, radiologists, psychologists, medical technicians, physical therapists, and other healthcare professionals.

### Evaluation of patients

Endocrine and metabolic evaluation included anthropometric measurements, evaluation of pubertal status assessed with Tanner staging (15), bone age and bone density measurement, lateral radiographs of the spine, and blood sampling for the determination of specific endocrine/metabolic parameters. Height (cm) and weight (kg) were measured using validated stadiometers (for ambulatory patients) and electronic digital scales, respectively. In non-ambulatory patients, segmental lengths in the recumbent position were measured by a trained medical professional using a non-rigid tape. The following segments were measured: from the top of the head to the right greater trochanter of the hip, hip to the right femoral epicondyle of the knee, and knee to the distal point of the calcaneus. Measurements were rounded to the first decimal place. Z-scores of height, weight, and body mass index (BMI) were calculated according to the Centers for Disease Control and Prevention (CDC) growth charts and the Coles LMS method (16). Bone mineral density and body composition were measured using dual-energy X-ray absorptiometry (using Hologic software, z-scores were not adjusted for height). Lateral spine X-rays were used to screen for the presence of vertebral fractures using Genant scoring (3, 17). Bone age was determined using the Greulich-Pyle method based on radiographs of the left wrist.

### Blood tests

Blood tests were performed in the morning following an overnight fast as part of the routine annual endocrinologic examination. The levels of growth factors (IGF-1, IGF-BP3), TSH, free T4, glucose, insulin, cholesterol (total cholesterol, HDL, LDL), triglycerides, and 25-hydroxy vitamin D3 were measured. Glucose was measured using the standard oxidase method (Beckman Coulter Glucose Analyzer, Beckman CoulterInc., CA, USA). Serum fasting insulin was measured by the two-site sandwich chemiluminescent immunoassay, and the Atellica IM Insulin (IRI) kit (AtellicaIM 1600 analyzer, Minaris Medical Co for Siemens Healthcare Diagnostics, USA). Total cholesterol and triglycerides were measured with enzyme method, LDL, and HDL with method elimination/catalysis (ADVIA^®^ Chemistry systems, Siemens Healthcare, Erlangen, Germany). For assessment of dyslipidemia, the reference ranges consistent with cut-off guidelines by the American Heart Association and the American Academy of Pediatrics were used (18). The insulin resistance was calculated with the Homeostatic Model Assessment for Insulin Resistance score (HOMA-IR) by the equation FPG (mmol/L) × FPI (mU/L)/22,5 (19). The HOMA-IR value was defined as normal/statistically increased IR based on the child’s sex and pubertal status (20).

The growth hormone (GH) axis was assessed by measuring insulin-like growth factor 1 (IGF-1) and insulin-binding protein (IGFBP3), together with height measurement and assessment of growth velocity. IGF-1 was measured by chemiluminescence using an SYS analyzer(IDS-iSYS Insulin-like Growth Factor-I Immunodiagnostic Systems Limited, Boldon, UK). If growth hormone deficiency was suspected, a GH stimulation test with arginine and levodopa was performed as a confirmation test. A peak GH value < 7ug/L in growing children was defined as GH deficiency (21). Hypogonadism was suspected when there were no signs of pubertal development (at the age of 15 years in boys) or pubertal arrest. Diagnosis was confirmed by performing a gonadotropin-releasing hormone test measuring LH, FSH, together with testosterone levels. Testosterone was measured using the radioimmunoassay method (Dia-Sorin S.p.A., Sallugia, Italy in Diagnostic Products Corporation, LA). Serum LH and FSH were measured by two-site sandwich, chemiluminescent immunoassay using the Atellica IM Luteinizing Hormone(LH) kit, Atellica IM Follicle Stimulating Hormone (FSH) kit, and Atellica IM 1600 analyzer (Minaris Medical Co for Siemens Healthcare Diagnostics, USA).

### Statistical analyses

Statistical analyses were conducted using the Statistical Package for the Social Sciences (SPSS 25). Owing to the nature of our study, descriptive statistics were used. Continuous variables were described using the mean and standard deviation. Categorical variables were described using frequencies.

## Results

During the study period, a total of 21 male patients were actively followed up at our center, and all were included in the study. The average age of the participants at the last annual follow-up was 11.1 years; the youngest patient in our cohort was 4.6 years of age, the oldest 16.7 years. Two of the youngest patients were corticosteroid-naive, and the rest were treated with a corticosteroid – deflazacort. None of the patients were treated with prednisolone. Mean duration of corticosteroid treatment was 5.4 years (range 1.1 - 11.3 years). None of the patients aged < 15 years (19/21) showed signs of pubertal development at the last annual multidisciplinary evaluation. In the two eldest patients, Tanner stage III was present at the time of the last annual evaluation. Due to slower progression of pubertal development and suspected arrest in development, they are being carefully monitored; at the time of writing none of them had been receiving testosterone replacement therapy. In one patient, growth hormone deficiency was diagnosed, and growth hormone replacement therapy was initiated. Cohort characteristics and the prevalence of endocrine and metabolic disorders are presented in **Table 1**.

**Table 1:** Characteristics and endocrine/metabolic status of the 21 boys followed at our center.

Characteristic	n (%)
Age (average, range)	11.1 (4.6-16.7) yrs
Ambulation	15/21 (71%)
CS therapy	19/21 (90%)
- Treatment duration	5.4 ± 2.6 yrs
- Deflazacort dose	0.65 ± 0.12 mg/kg
Delayed pubertal development/arrest*	2/2
Testosterone therapy	0
Antropometrics	
Height SDS	-2.48 ± 1.29
- Short stature (Height SDS ≤ -2)	14/21 (67%)
Weight SDS	-0.47 ± 1.20
BMI SDS	1.20 ± 1.26
- BMI SDS ≥ 2	6/21 (29%)
Body fat % SDS	2.49 ± 0.73
- Body fat % ≥ 2 SDS	12/21 (57%)
Bone health	
DXA Z-score WB	-3.76 ± 2.01
- DXA Z-score WB ≤ -2	15/20 (75%)
DXA Z-scores L	-1.62 ± 0.90
- DXA Z-score L ≤ -2	8/20 (40%)
Pathologic fracture	10/21 (48%)
- Vertebral/long bone fractures	9/1
Vitamin D levels [range]	62.7 nmol/L [40–91 nmol/L]
D vitamin supplement	20/21 (95%)
Calcium supplement	16/21 (76%)
Bisphosphonate therapy	10/21 (48%)
Metabolic health	
HOMA-IR	1.93 ± 1.65
Insulin resistance	2/21 (10%)
- Any lipid abnormality**	16/21 (76%)
Increased TC (≥ 4.4 mmol/L)	13/21 (62%)
- Increased LDL (≥ 2.8 mmol/L)	10/21 (48%)
- Increased TG (≥ 1.7 mmol/L)	7/21 (33%)
- Hypertension	0

*in those old enough to assess: boys aged 15 years or more.

**increased defined as borderline category for specific age group (18).

BMI, Body Mass Index; CS, Corticosteroid; DXA WB, Dual-energy x-ray absorptiometry whole body; DXA L, Dual-energy x-ray absorptiometry lumbar region; HOMA-IR, Homeostatic Model Assessment for Insulin Resistance score; LDL, Low-Density Lipoprotein; SDS, Standard Deviation Score; TC, Total Cholesterol; TG, Triglycerides.

**Figure 1:**
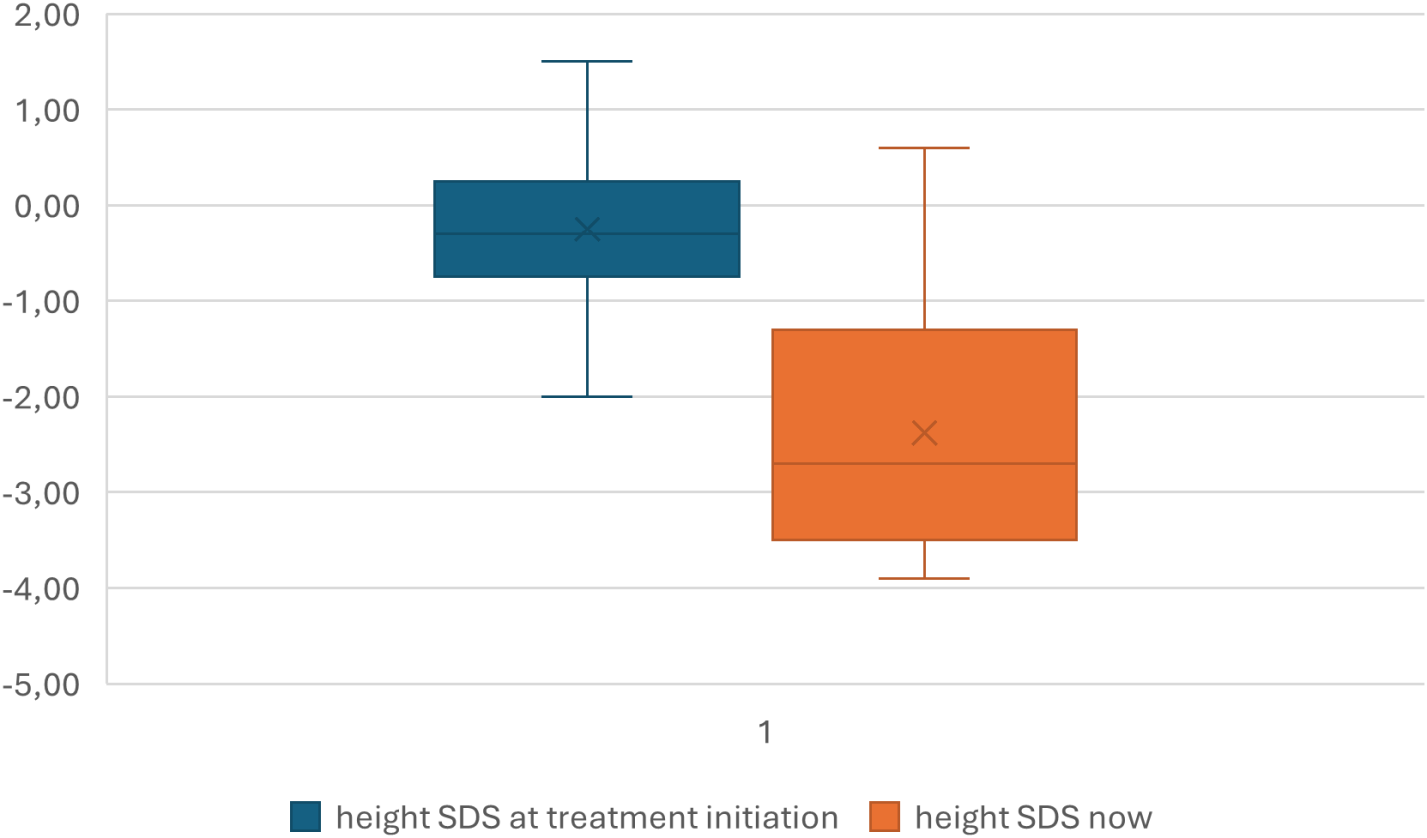
Change of height SDS since the corticosteroid treatment initiation.

Short stature, defined as a height standard deviation score (SDS) ≤ -2 for the child’s age and sex, was present in 67% of DMD patients at the last annual follow-up. A decline in height SDS since the initiation of corticosteroid therapy was observed, with -2.37 SDS on average (**Figure 1**, **Table 2**). Obesity, defined as a BMI SDS ≥ 2 for the child’s age and sex, was present in 29% of the cohort. Based on body composition data (fat% SDS), 57% of the participants were classified as obese. Lipid abnormalities were present in 76% of the patients. No hypertension was observed. Insulin resistance, defined based on the HOMA-IR calculation, was present in 9.5% of the patients.

**Table 2:** Linear growth and bone density throughout the years.

Patients	Age (yrs)	Ambulation	CS treatment duration	Height SDS at last follow-up	Δ height SDS (since CS initiation)	Bone age SDS (GP method*)	Bisphosphonate therapy	DXA Z-score L	Δ Z-score L (in the last year)	DXA Z-score WB	Δ Z-score WB (in the last year)
1	4.6	Yes	n/a	-0.4	n/a	-1.4		n/a	n/a	n/a	n/a
2	5.2	Yes	1.1	0.6	-0.6	0.2		-0,7	0.2	n/a	n/a
3	6.5	Yes	n/a	-1.0	n/a	-0.5		-0.7	0.2	-1.3	-0.5
4	8.4	Yes	2.1	-1.0	-1.0	0.4		-0.9	-0.3	-2.5	-0.7
5	9.0	Yes	2.9	-1.9	-1.6	-0.6		-1.3	0	-2.5	-0.2
6	9.1	Yes	3.7	-3.7	-1.9	-1.2	Yes	-1.9	0.4	n/a	n/a
7	10.0	Yes	5.5	-3.9	-3.3	-2.2	Yes	-3.0	-0.4	-4.6	-0.9
8	10.1	Yes	4.0	-2.7	-2.5	-1.1	Yes	-2.4	0.5	-3.0	-0.4
9	10.2	Yes	5.7	-2.5	-1.8	-1.9		-0.4	-0.2	-1.3	-0.2
10	10.6	Yes	3.5	-0.8	-0.7	0.4		-1.5	-0.6	-1.0	-0.3
11	10.7	Yes	4.0	-3.5	-1.5	-2.1		-1.5	-0.8	-3.4	-1.4
12	11.5	Yes	4.0	-1.6	-1.0	-1.4		-1.0	0.4	-2.7	-0.3
13	12.8	No	6.7	-3.5	-3.2	-5.6	Yes	-2.2	0.4	-5.6	n/a
14	13.1	No	5.1	-1.6	-0.9	-0.6		-2.7	-0.1	n/a	n/a
15	13.2	No	4.5	-3.5	-2.4	-4.4	Yes	-1.2	0.7	-4.5	-0.1
16	13.2	Yes	4.7	-3.1	-3.0	-2.3	Yes	-2.8	0.1	-6.5	-0.4
17	13.6	No	8.0	-2.9	-3.6	-3.5	Yes	-0.7	-1	-3.7	-0.4
18	13.9	Yes	7.0	-3.2	-3.4	-3.3	Yes	-2.3	-0.5	-3.5	-0.6
19	14.5	Yes	8.2	-2.3	-3.8	-0.9		-2.2	-0.4	-2.0	-1.5
20	15.9	No	11.3	-3.6	-4.8	-1.9	Yes	-1.8	0.2	-6.2	-0.7
21	16.7	No	10.0	-3.8	-4.1	-3.3	Yes	-2.0	0	-8.9	-1

*Greulich Pyle method.

CS, corticosteroid; SDS, Standard Deviation Score; DXA WB, Dual-energy x-ray absorptiometry whole body; DXA L, Dual-energy x-ray absorptiometry lumbar region.

Patient No colored in gray: potential benefit from switch to vamorolone.

Low bone mineral density in the lumbar region and the whole body (defined by Z-score ≤ -2) was detected in 40% and 75% of DMD patients, respectively. The decline in Z-scores in the last year was progressive; from a mean value of -1.33 to -1.62 ± 0.90 in the lumbar region and -2.31 to -3.76 ± 2.01 for the whole body (**Table 2**). Ten patients (48%) had a pathological fracture during their lifetime; vertebral fractures were identified in nine patients, while one patient sustained a long bone fracture. In all treatment with intravenous bisphosphonates (zolendronic acid) was initiated. The mean vitamin D level at the time of the last annual check-up was 62.4 nmol/L (range 40–91 nmol/L); and vitamin D supplement was prescribed to the majority of patients (95%). Calcium supplement was prescribed to 76% of the cohort. In the two corticosteroid-naïve patients, none of the above endocrine/metabolic complications were observed.

## Discussion

In our national cohort of pediatric patients with DMD, we found at least one endocrine and/or metabolic disorder affecting growth, pubertal development, bone health, and/or lipid and glucose metabolism in 19 out of 21 patients. Furthermore, we have identified 11/21 patients who could potentially benefit from a therapeutic switch from deflazacort to vamorolone.

Our findings on impaired linear growth, with two-thirds of our cohort being short-statured, are concordant with the previously published reports, describing short stature and progressive decline in growth as a corticosteroid treatment-related side effect (22–25). Additionally, natural history studies in glucocorticoid-naïve DMD patients have shown slower growth apparent already in early childhood, leading to reduced adult height by one standard deviation (26). The degree of growth suppression varies among reports and it is reported to be less pronounced in intermittent glucocorticoid use. The use of deflazacort has been associated with a bigger decline in growth trajectory compared to prednisone (27). One patient in our cohort was diagnosed with growth hormone deficiency (in adolescence), and treatment with growth hormone has recently been initiated. According to the guidelines, growth hormone treatment is not indicated for the treatment of all patients with DMD, but is reserved for those who have a clear deficiency of growth hormone (3).

Signs of pubertal development were present in the two eldest patients; however, at the last follow-up visit, pubertal arrest was suspected in both patients and is currently being investigated. Hypogonadotropic hypogonadism represents another anticipated adverse effect of glucocorticoid therapy and has been consistently documented in the majority of adolescents with DMD undergoing daily glucocorticoid treatment (27, 28). The latest guidelines suggest starting testosterone therapy at the onset of pubertal delay (≥14 years of age), considering it already at 12 years of age in the case of receiving corticosteroids and the absence of pubertal development. No DMD-specific guidelines for the timing of pubertal induction are currently available (3). With the testosterone treatment, positive effects on bone metabolism and growth are expected (27, 29). Additionally, positive psychosocial aspects of treatment have recently been described by Sodero et al., and should outweigh the potential risks of treatment (7). Accordingly, careful monitoring of pubertal development starting in boys aged 12 years is essential to ensure timely therapeutic intervention and prevent the undertreatment of hypogonadism.

The combined effects of glucocorticoid-induced myopathy and osteotoxicity contribute to another commonly observed complication in DMD — osteoporosis. It is estimated that 20–60% of affected boys experience low-trauma extremity fractures during their lifetime (4). The reported prevalence of both symptomatic and asymptomatic vertebral fractures in patients treated with deflazacort exceeds 50% and increases with the duration of therapy (30). Our findings are consistent with these reports, demonstrating a vertebral fracture prevalence of 47% (9/19) after a similar period of deflazacort treatment.

About a third of our cohort was classified as obese based on the BMI SDS calculation; however, based on the BMI measurement alone, the prevalence of obesity was likely underestimated as in terms of body composition, it was present in 57%. The prevalence of obesity in our cohort is comparable to that reported in other observational studies, in which the BMI Z-score was likewise found to be an inadequate indicator of obesity (31, 32). Due to the nature of the disease, individuals with DMD have a higher percentage of body fat and a lower percentage of lean body mass than healthy subjects. The increase in fat mass is progressive, as a consequence of the loss of muscle tissue and progressive intramuscular fat deposition, which occurs particularly with the loss of ambulation (31, 33). Weight gain in DMD patients is another expected adverse effect of corticosteroid therapy. The treatment with vamorolone has shown a similar increase of BMI to that observed with prednisolone (13).

Metabolic abnormalities such as dyslipidemia and insulin resistance were present in 76% of the cohort. Observed abnormalities can be secondary to corticosteroid treatment; however, recent findings increasingly consider DMD a new form of primary dyslipidemia, a systemic metabolic disease known to affect cholesterol and triglyceride levels (34, 35). Abnormalities in lipid metabolism are known to cause pathological changes in skeletal muscle tissues, such as the loss of muscle mass, density, and strength. Additionally, the disruption of lipid metabolism is also related to increased insulin resistance, sarcopenia, and diabetes (36). The effect of dyslipidemia on muscle wasting in DMD still needs to be clarified; however, emerging data support this concept (35).

### Selection of patients with endocrine/metabolic comorbitities for switch to vamorolone treatment

Since December 2023, vamorolone, in addition to prednisolone and deflazacort, has been approved as a treatment option for DMD (9) in Europe, and became available in Slovenia in April 2024. In short-term follow-up studies (24 weeks), its use was associated with retained motor function, compared with prednisolone, while reducing some of the side effects affecting bone health and linear growth (37). To date, no study has compared vamorolone with deflazacort. Longer follow-up data (>30 months), treatment effects on non-ambulatory patients, effects on pubertal development, metabolic effects, or cataract development are not yet known (13). The use of vamorolone is associated with an increase in BMI similar to that of prednisolone (13). Similar to other corticosteroids, it causes adrenal suppression and requires hydrocortisone substitution in patients with acute illness (38). Recently, the antagonistic effect of vamorolone on mineralocorticoid receptors was confirmed in humans, which could result in an important cardioprotective function in DMD (11). However, evidence supporting the beneficial effects of vamorolone on cardiac hypertrophy and fibrosis remains limited to preclinical studies at present.

The price of vamorolone is substantially higher than that of the other two corticosteroids available on the market (39), presenting a higher financial burden for an individual or insurance company. As the demand and expectations of the patients’ caretakers are high and the data on the potential benefits of vamorolone are limited, we attempted to determine the characteristics of a subpopulation of DMD patients that would benefit most from the new treatment available.

Based on the relatively short-term available data, there are two favorable endocrine effects of vamorolone use: improved bone health and linear growth. Regarding bones, there was lower suppression of bone turnover markers compared to prednisolone use (osteocalcin, P1NP, and CTX) and a reduction in vertebral fracture rates (follow-up period of 2.5 years) (12). Regarding linear growth, no change in growth velocity or decrease in height Z-score was observed within 48 weeks of vamorolone use. Catch-up growth was noted in patients who switched from prednisolone to vamorolone (13). There are no follow-up data available on the possible effects of taller stature on preserving motor function and the incidence of scoliosis. Theoretical concerns of the detrimental impact of taller stature on muscle function in DMD have already been raised (24, 40).

Based on the available data of vamorolone use on its potential beneficial effects on bone health and linear growth, we suggest the following criteria for therapy switch to vamorolone **Table 3**. Considering its higher cost compared to deflazacort/prednisone we suggest it only as a switch therapy to the non-corticosteroid naive ambulatory DMD patients with corticosteroid-induced side effects affecting bone health and linear growth. Currently, the beneficial effects of vamorolone use have been described only in ambulatory DMD patients. Therefore, the switch was offered only to those with preserved ambulation. For transition from deflazacort the patient has to fulfill at least one of the following criteria: presence of pathological fracture (vertebral or long bone), DXA Z-scores with lumbar and/or whole-body region Z-score ≤ -2, short stature (height SDS ≤ -2), or decline of growth trajectory defined as height SDS decrease of ≥ 1.3 after corticosteroid treatment initiation (with preserved growth potential based on the bone age measurement) (41).

**Table 3:** Suggested criteria for switch to vamorolone.

Indications for vamorolone treatment in ambulatory non corticosteroid-naive DMD patients	
Bone	• history of pathological fracture(s); vertebral or long bone • lumbar and/or whole-body region DXA Z-score ≤ -2
Growth	• short stature (height SDS ≤ -2) • decline in growth trajectory; height SDS decrease of ≥ 1.3 after corticosteroid treatment initiation (if there is preserved growth potential based on the bone age measurement)

SDS, Standard Deviation Score; DXA, Dual-energy x-ray absorptiometry.

We have thus identified 11 ambulatory patients previously receiving deflazacort (patients Nr. 4,5,6,7,8,9,11,12,16,18 and 19), who could potentially benefit from a switch to vamorolone (marked in **Table 2**). As growing evidence emerges regarding the long-term safety and systemic effects of vamorolone, we anticipate that our current recommendations for therapy modification will require revision. Future adjustments to the clinical criteria will be directed toward optimizing therapeutic efficacy while ensuring the judicious allocation of healthcare resources.

Our study and our clinical considerations are limited by the number of DMD patients in our country. In Slovenia, currently, all the patients are treated with deflazacort, therefore we cannot compare treatment efficacy with other corticosteroids, i.e. prednisolone. Future studies on a larger number of patients are needed to evaluate the long-term safety and efficacy of vamorolone across diverse patient subgroups, including non-ambulatory individuals, and to clarify its impact on puberty, cardiac function, metabolic health, and other organ systems. Careful patient selection based on endocrine and metabolic risk profiles may help optimize treatment outcomes and quality of life for patients with DMD, while rationally using financial resources available for novel treatment options for DMD patients.

## Conclusion

Our national cohort study confirmed a high prevalence of endocrine and metabolic complications among pediatric patients with Duchenne muscular dystrophy (DMD), particularly short stature, absent or arrested pubertal development, low bone mineral density, and dyslipidemia. These findings highlight the substantial burden of corticosteroid-associated comorbidities in this population.

Given the availability of vamorolone in European countries, including Slovenia, we suggest clinical criteria to identify patients who may benefit from switching from deflazacort to vamorolone based on the currently available data on potential beneficial effects. We propose that non-corticosteroid naive ambulatory DMD patients with pathological fractures, significantly reduced bone density, short stature, or significant growth deceleration could be considered for this switch. Patients undergoing a switch to vamorolone should continue to be carefully monitored (we plan to evaluate them every 3 months within the first year) to evaluate the treatment effect and detect possible adverse events. Follow-up data from our cohort will provide evidence to support or refute this approach.

## Data Availability

The original contributions presented in the study are included in the article/supplementary material, further inquiries can be directed to the corresponding author/s.
